# Quality in Acute Stroke Care (QASC) Germany: improving efficiency in stroke care with nurse-initiated FeSS-protocols

**DOI:** 10.1186/s42466-024-00352-1

**Published:** 2024-10-17

**Authors:** Anne-Kathrin Cassier-Woidasky, Sandy Middleton, Simeon Dale, Kelly Coughlan, Catherine D’Este, Elizabeth McInnes, Dominique A. Cadilhac, Waltraud Pfeilschifter

**Affiliations:** 1 Saarland University of Applied Sciences, htw saar, Goebenstraße 40, Saarbrücken, D-66117 Germany; 2 Nursing Research Institute, St Vincent’s Health Network Sydney, St Vincent’s Hospital Melbourne and Australian Catholic University, Sydney, Australia; 3 School of Nursing, Midwifery and Paramedicine, Australian Catholic University, Sydney, Australia; 4 The Sax Institute, Sydney, Australia; 5 School of Medicine and Public Health, University of Newcastle, Sydney, Australia; 6 Translational Public Health Division, Stroke and Ageing Research, School of Clinical Sciences, Monash University, Melbourne, Australia; 7 Public Health, Stroke Division, The Florey Institute of Neuroscience and Mental Health, University of Melbourne, Melbourne, Australia; 8 Department of Neurology and Clinical Neurophysiology, Klinikum Lueneburg, Lueneburg, Germany

**Keywords:** Stroke unit, Nurse-led intervention, FeSS protocol, Fever, Hypergycaemia, Dysphagia, Nurses

## Abstract

**Background:**

Nurse-initiated supported implementation of protocols to manage fever, hyperglycaemia (sugar) and swallowing (FeSS) following acute stroke reduced 90-day death and disability in the landmark Australian Quality in Acute Stroke Care (QASC)-Trial. An international interprofessional collaboration sought to evaluate the effects of nurse-led FeSS implementation on FeSS Protocol adherence in German stroke units.

**Methods:**

This pre-test/post-test study was conducted in eight German stroke units between 2020 and 2022. Stroke nurses as clinical champions, supported by the project team, conducted multidisciplinary workshops discussing pre-implementation medical record audit results, barriers and facilitators to FeSS Protocol implementation, developed action plans and provided education, with ongoing support from Australia. Medical record audit data were collected by nurses, pre-implementation and three months post-implementation.

**Results:**

In 771 (pre-implementation) and 679 (post-implementation) patients there were improvements in overall FeSS adherence (pre 20%, post 28%; adjusted difference in proportions (95% CI) 11%, (5.1%, 16%); *p* < 0.001), adherence to hyperglycaemia (pre 43%, post 55%; adjusted difference 23%, (17%, 29%); *p* < 0.001) and swallowing (pre 52%, post 61%; adjusted difference 11%, (5.2%, 17%); *p* < 0.001) but not fever protocol (pre 76%, post 78%; adjusted difference 1.5%, (-2.6%, 5.7%); *p* = 0.474). Improvements also were noted in administration of anti-pyretics (pre 29%, post 59%; adjusted difference 32%, (20%, 44%); *p* < 0.001); and insulin (pre 41%, post 60%; adjusted difference 14%, (1.1%, 28%); *p* < 0.034) both within one hour, as well as in performing a swallow screen within 24 h of admission (pre 65%, post 74%; adjusted difference 18% (8.8%, 26%); *p* < 0.001).

**Conclusions:**

Supported implementation of the FeSS Protocols significantly improved acute care for post stroke complications of fever, hyperglycaemia and dysphagia in terms of higher adherence and shorter time to treatment.

**Trial registration:**

As this is a pre-test/post-test study and does not meet the WHO/ICMJE definition of a clinical trial, registration was not required.

## Background

Cornerstones of stroke therapy are monitoring and treatment in a stroke unit by an interprofessional team [1], with the aim of quickly identifying and treating frequent complications such as fever, hyperglycaemia, dysphagia (or FeSS: **Fe**ver, **S**ugar, **S**wallowing) and pneumonia [2]. In practice, measurement of physiologic parameters and treatment in cases of deviations from the target ranges is a fragmented process [3], which is a barrier for the quality of care. Vital parameters are measured by nurses [4]; to treat deviations in Germany requires nurses to consult with a physician potentially causing avoidable delays. Similar delays, contributing to extended ‘nil-by-mouth’ times for patients, may exist when a specialist referral is made for a swallow assessment. This can be prevented by using FeSS Protocols as a mandatory **s**tandard **o**perating **p**rocedure (SOP), which bestow the responsibility for stroke care upon nurses within defined limits. In Australian stroke units, supported implementation of the nurse-led FeSS Protocols resulted in reduced 90-day mortality and dependence [5], and reduced mortality over a median of four years [6]. Importantly, and contrary to recanalizing therapies with specific eligibility criteria, all patients with ischemic stroke and intracerebral hemorrhage benefit from standardized stroke unit care [7]. The FeSS Protocols are now part of the clinical guidelines for stroke management in Australia (informme.org.au), with international implementation of these protocols more recently into 64 hospitals in 17 European countries [8].

In Germany, there is a well-established system of certified stroke units, highly recognized guidelines on treatment [9], and specifications of the treatment of patients with stroke as defined in the German “Operationen und Prozeduren Schlüssel” OPS (OPS 8-981) [10]. As the OPS documentation is mandatory for organizational remuneration, we hypothesized that this system promises to ensure a high level of adherence to the basic stroke care measures. However, by transferring responsibility and competencies to nurses to measure and treat the basic vital parameters of stroke using the FeSS Protocols we expected improved treatment (in terms of the percentage of patients with out-of-range parameters that actually received treatment), and shorter time intervals from measurement to treatment for deviated values of body temperature and glucose and swallowing. We aimed to evaluate the effects of nurse-led FeSS implementation on FeSS Protocol adherence in German stroke units.

## Methods

**Fig. 1:**
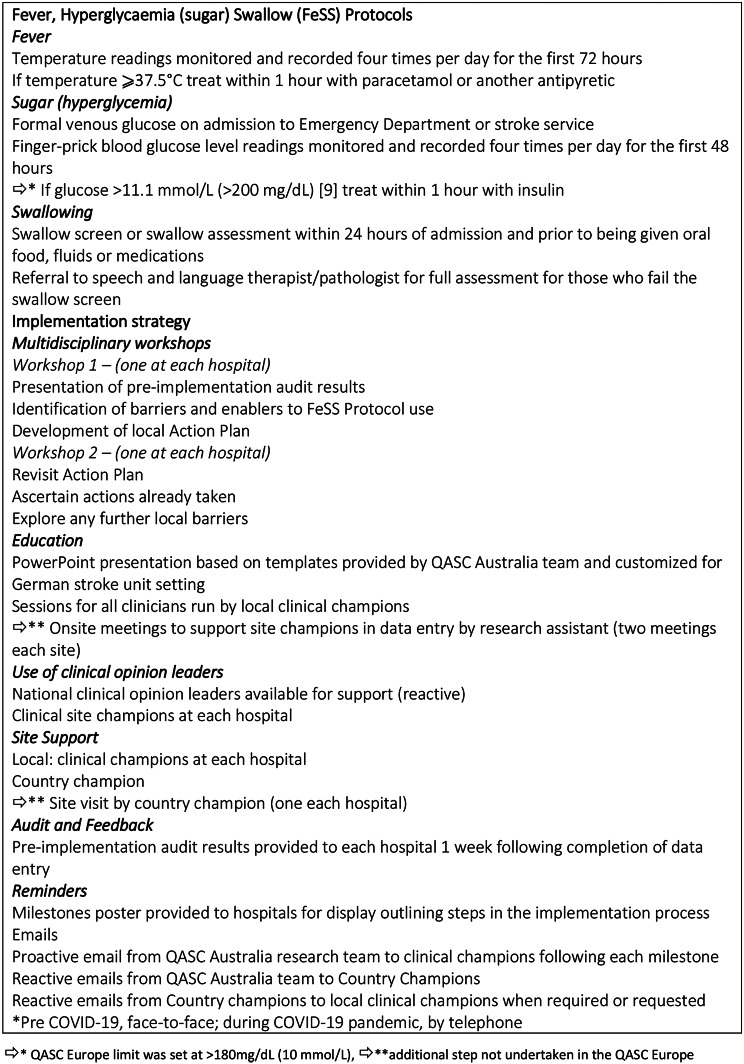
QASC Germany intervention component, based on QASC Europe intervention [8]

This was a single-country, multicenter study, implementing an intervention with a pre-test/post-test evaluation as described in Middleton et al. [8] (Fig. 1). We implemented the FeSS Protocols according to the Quality in Acute Stroke Care (QASC) Europe project [8]. The intervention was the implementation of the FeSS Protocols as a standard operating procedure (SOP) providing the legal framework to handover monitoring and management of the stroke related basic measures to nurses, including the transfer of responsibilities to implement the guideline-based basic stroke care from a physician centered medical model to a nurse-led model in collaboration with the medical staff [8]. Clinical data were routinely documented in patient records as part of stroke diagnosis and therapy as well as the OPS documentation required for remuneration.

### Setting

This study was carried out at eight stroke units certified according to the criteria of the German Stroke Society (DSG) that are part of a regional interdisciplinary neurovascular network [11, 12]. Patient eligibility criteria were: discharge diagnosis of ischemic stroke or intracerebral hemorrhage, presentation to the hospital within 48 h of stroke onset and not receiving end-of-life care.

## Nurse-led implementation

The QASC intervention [8] was adapted slightly to local guidelines, marked with ⇨in Fig. 1 (Fig. 1). The German FeSS Protocol has been approved by the Director of the Department of Neurology in the leading University Hospital [13]. The evidence-based FeSS-SOP [13] was implemented through central workshops and local practice discussions according to Middleton et al. [8] as outlined in detail in Fig. 1.

### Outcome measures

The primary outcome was binary measure of adherence with all monitoring and treatment elements of the FeSS Protocols as recorded in the medical records (composite measure). Secondary outcomes were the adherence to each of the combined monitoring and treatment elements for: (i) fever, (ii) hyperglycaemia and (iii) swallowing. The tertiary outcomes were adherence to the individual elements of the FeSS Protocols (Fig. 1).

### Data collection

Pre-implementation data were collected retrospectively from 60 to 100 consecutive patient charts according to stroke unit case load at each of the participating stroke units by trained stroke unit ward nurses (“site champions”) in accordance with the QASC Europe study. Stroke units with > 800 annual cases were required to enter 100 patient records; those with < 800 annual cases were required to enter 60 records. Pre-implementation data collection for consecutive acute stroke patients treated from September to December 2019 took place from June to August 2020. Post-implementation, prospective, data were collected in the corresponding months one year later using identical methods to those used in the pre-implementation data collection phase. Post-implementation data were collected from 1 September 2021, until the target sample size was reached. All data were extracted from patient records and entered pseudonymized into a database hosted by the QASC Australia team. As in the QASC Europe project, nurses were trained to extract data and to introduce the FeSS Protocols, supported by the national research team and the QASC Australia team.

### Statistical analyses

Analyses were undertaken using the R and Stata statistical packages using methods consistent with the QASC Europe study to enable comparison of results. Patient demographic and clinical characteristics were compared for pre and post-implementation patients using the chi square test or Fisher’s exact test. Time to paracetamol and insulin administration was calculated on the first elevated measure as documented in the medical records.

Pre- to post-implementation change in outcomes were assessed separately for each primary, secondary and tertiary outcome using mixed effects logistic regression which included variables for implementation status (pre or post), age group (18–64, 65–74, 75–84, 85+), sex and stroke severity (NIHSS) and adjusted for correlation of outcomes within hospital. Adjusted differences in proportions from pre- to post-implementation are reported with 95% Confidence Interval (CIs), estimated as average margin effects using the dydx option for the margins command in Stata. Observations with missing values for covariates were excluded from the regression models (complete case analyses) but are included in descriptive tables where appropriate.

## Results

Out of 15 stroke units in the INVN Rhein-Main network, eight stroke units agreed to participate. A total of 1450 patients from eight stroke units were included in the study: 771 in the pre-implementation audit and 679 in the post-implementation audit.

### Pre-implementation characteristics

Demographic characteristics and premorbid risk factors were generally similar in the pre- and post-implementation cohorts (Table 1), except for higher pre-implementation levels of obesity (pre 10% vs. post 4.6%, *p* < 0.001), pre-stroke disability (pre-morbid modified Rankin Score [mRS]) > 1 (pre 49% vs. post 37%, *p* < 0.001). Fewer patients received endovascular stroke therapy (EVT) in the post-implementation cohort (pre 11% vs. post 7.0%, *p* = 0.014), and more patients receiving EVT in the post-implementation cohort underwent non-general anesthesia (8.8% vs. 30%, *p* = 0.003).

**Table 1:** Pre-implementation characteristics of all patients

Characteristic	Level	Pre 771 *n* (%)	Post 679 *n* (%)	*p*-value^
Age group (years)	18 to 64	196 (25%)	164 (24%)	0.270
	65 to 74	167 (22%)	148 (22%)	
	75 to 84	265 (34%)	214 (32%)	
	85+	141 (18%)	151 (22%)	
Gender	Male	420 (55%)	386 (57%)	0.359
	Female	349 (45%)	291 (43%)	
Time from onset of symptoms to hospital admission (mins)	median (Q1, Q3)	170 (84, 450)	154 (77, 413)	0.437
Able to walk unassisted on admission	Yes	377 (53%)	339 (53%)	0.984
	No	334 (47%)	301 (47%)	
Premorbid risk factors	Stroke	155 (20%)	139 (20%)	0.862
	Diabetes	190 (25%)	161 (24%)	0.679
	Hypertension	548 (71%)	504 (74%)	0.180
	History of smoking	114 (15%)	88 (13%)	0.316
	Obesity (BMI ≥ 30)	77 (10%)	31 (4.6%)	**< 0.001**
	None of the above	126 (16%)	110 (16%)	0.942
NIHSS recorded on admission	Yes	675 (88%)	664 (98%)	**< 0.001**
National Institute of Health Stroke Scale (NIHSS)	0 to 7 (mild stroke)	510 (76%)	482 (73%)	0.204
	8 to 16 (moderate stroke)	124 (19%)	149 (22%)	
	17+ (severe stroke)	36 (5.4%)	33 (5%)	
Premorbid modified Rankin Score	0 (No symptoms at all)	234 (30%)	323 (48%)	**< 0.001**
	1 (No significant disability despite symptoms)	155 (20%)	103 (15%)	
	2 (Slight disability)	114 (15%)	101 (15%)	
	3 (Moderate disability)	146 (19%)	92 (14%)	
	4 (Moderately severe disability)	81 (11%)	39 (5.8%)	
	5 (Severe disability)	40 (5.2%)	16 (2.4%)	
Premorbid modified Rankin Score (dichotomised)	mRS > 1	381 (49%)	248 (37%)	**< 0.001**
	mRS ≤ 1	389 (51%)	426 (63%)	
Stroke type	Ischemic Stroke	694 (90%)	616 (91%)	0.510
	Intracerebral haemorrhage	72 (9.4%)	55 (8.1%)	
	Undetermined	4 (0.52%)	6 (0.89%)	
Received intravenous thrombolysis	Yes	226 (29%)	176 (26%)	0.155
	No	544 (71%)	501 (74%)	
Received endovascular therapy	Yes	82 (11%)	47 (7.0%)	**0.014**
	No	687 (89%)	629 (93%)	
ECR anaesthesia	Under general anaesthesia	73 (91%)	28 (70%)	**0.003**
	Under non-general anaesthesia	7 (8.8%)	12 (30%)	

^p-values from Pearson Chi-Squared test for categorical variables and Wilcoxon rank sum test for continuous variables; P values not adjusted for correlation within sites; Bold indicates p values < 0.05; numbers may not add to total sample size due to missing values and rounding

Consistent with higher rates of pre-stroke disability in the pre-implementation cohort, there were more patients with relevant disability (mRS > 1) at discharge (Table 2) in the pre-implementation (pre 63% vs. post 55%, *p* = 0.004). However, discharge patterns varied in the pre- and post-implementation cohorts (*p* < 0.001); 55% of patients pre- and 45% post-implementation were discharged home, with 34% pre and 42% post transferred to inpatient rehabilitation) and 6.2% pre vs. post 9.3% transferred to a nursing home. Hospital (but not stroke unit) stay was longer in the post-implementation group (pre; median 171; [Quartile 1 (Q1), Quartile 3 (Q3): 120, 266] vs. post median 194; [Q1, Q3: 139, 285] hours; *p* < 0.001) (Table 2).

**Table 2:** Outcome measures

Outcome	Level	Pre 771 n (%)	Post 679 n (%)	*p*-value^
Developed pneumonia during stroke unit stay	Yes	34 (4%)	42 (6%)	0.132
	No	736 (96%)	637 (94%)	
Able to walk unassisted on discharge	Yes	511 (68%)	470 (70%)	0.295
	No	244 (32%)	199 (30%)	
Discharge destination	Home	402 (55%)	303 (45%)	**< 0.001**
	Rehabilitation facility	251 (34%)	285 (42%)	
	Long term care facility/nursing home	46 (6%)	63 (9.3%)	
	Other hospital	26 (3.5%)	13 (1.9%)	
	Deceased	12 (1.6%)	13 (1.9%)	
Length of stroke unit stay (hrs)	median (Q1, Q3)	89 (75, 121)	90 (75, 121)	0.587
Length of hospital stay (hrs)	median (Q1, Q3)	171 (120, 266)	194 (139, 285)	**< 0.001**
NIHSS recorded on discharge	Yes	376 (49%)	471 (70%)	**< 0.001**
NIHSS at discharge	0 to 7 (mild stroke)	329 (88%)	409 (87%)	0.697
	8 to 16 (moderate stroke)	39 (10%)	54 (11%)	
	17+ (severe stroke)	8 (2.1%)	7 (1.5%)	
Discharge mRS	0 (No symptoms at all)	115 (15%)	182 (27%)	**< 0.001**
	1 (No significant disability despite symptoms)	170 (22%)	119 (18%)	
	2 (Slight disability)	111 (14%)	113 (17%)	
	3 (Moderate disability)	156 (20%)	98 (14%)	
	4 (Moderately severe disability)	108 (14%)	64 (9.5%)	
	5 (Severe disability)	97 (13%)	87 (13%)	
	6 (Deceased)	12 (1.6%)	13 (1.9%)	
Discharge mRS (dichotomised)	mRS > 1	484 (63%)	375 (55%)	**0.004**
	mRS ≤ 1	285 (37%)	301 (45%)	

^p-values from Pearson Chi-Squared test for categorical variables and Wilcoxon rank sum test for continuous variables; P values not adjusted for correlation within sites; Bold indicates P values < 0.05; numbers may not add to total sample size due to missing values

### FeSS management

#### Primary outcome

A statistically significantly larger proportion of patients received all the management and treatment elements of the FeSS Protocols from pre-to-post implementation (pre 20%, post 28%; adjusted difference in proportions (95% CI) 11% (5.1%, 16%); *p* < 0.001) (Table 3).

#### Secondary outcomes

There was no change from pre-to-post implementation for care adhering to all the combined elements of the fever protocol (pre 76%, post 78%; adjusted difference in proportions (95% CI) 1.5% (-2.6%, 5.7%); *p* = 0.474). A significantly greater proportion of patients from pre-to-post implementation received care adhering to all the combined elements of hyperglycaemia protocol (pre 43%, post 55%; adjusted difference in proportions (95% CI) 23% (17%, 29%); *p* < 0.001), as well as for care adhering to all the combined swallowing elements (pre 52%, post 61%; adjusted difference in proportions (95% CI) 11% (5.2%, 17%); *p* < 0.001) (Table 3).

#### Tertiary outcomes

There were statistically significant improvements from pre-to-post implementation in the proportion of patients with elevated temperature ≥ 37.5 C given Paracetamol (pre 35% vs. post 68%; adjusted difference in proportions (95% CI) 33% (20%, 45%); *p* < 0.001), those with elevated temperature given Paracetamol within one hour from elevated temperature (pre 29% vs. post 59%; adjusted difference in proportions (95% CI) 32% (20%, 44%); *p* < 0.001); having a venous blood glucose level (BGL) sample taken (pre 82% vs. post 85%; adjusted difference in proportion (95% CI) 8.1% (1.5%, 15%); *p* = 0.016); monitoring of BGL on day 1 (pre 77% vs. 78%; adjusted different in proportions (95% CI) 5.7%, (1.6%, 9.9%); *p* = 0.007) and day 2 (pre 53% vs. post 70%; adjusted difference in proportions (95% CI) 20% (12%, 27%); *p* < 0.001); the proportion of patients who received insulin within one hour of elevated BGL (pre 41% vs. post 60%; adjusted difference in proportions (95% CI) 14% (1.1%, 28%); *p* = 0.034); receiving a formal swallow screen (pre 69% vs. post 75%, adjusted difference in proportions (95% CI) 15% (5.5%, 24%); *p* = 0.002) and receipt of a swallow screen within 24 h of admission to hospital (pre 65% vs. post 74%; adjusted difference in proportions (95% CI) 18% (8.8%, 26%); p = < 0.001 (Table 3). There were no other pre-to-post implementation changes in the individual elements of FeSS Protocols.

**Table 3:** FeSS Management pre- vs. postimplementation

		Pre n (%)	Post n (%)	Adj diff (%), (95% CI)	p-value^
**Patient records entered**		**771 (100)**	**679 (100)**	**-**	**-**
*Monitored and treated according to all FeSS Protocols (composite score)* ^#^		129 (20)	186 (28)	11 (5.1, 16)	**< 0.001**
***Temperature monitoring and treatment***					
Temperature monitored at least four times per day	Day of admission^1^	752 (98)	637 (94)	-2.31 (-5.5, 0.9)	0.156
	Day two of admission^1^	758 (98)	664 (98)	-0.03 (-1.4, 1.4)	0.970
	Day three of admission^1^	737 (96)	620 (92)	-3.1 (-6.7,0.5)	0.092
Temperature ≥ 37.5 °C recorded within 72 h of admission		205 (27)	159 (23)	-3.1 (-7.7, 1.5)	0.192
Paracetamol (or other anti-pyretic) given for first temperature ≥ 37.5 °C		71 (35)	106 (68)	33 (20, 45)	**< 0.001**
Paracetamol (or other anti-pyretic) given with one hour from first temperature ≥ 37.5°C^1^		58 (29)	92 (59)	32 (20, 44)	**< 0.001**
Time from first temperature ≥ 37.5 °C to anti-pyretic administration, mins (Median, (Q1, Q3))		15 (1.5,60)	10 (0,35)	-	0.2354
Monitored and treated according to the Fever Protocol^1^		582 (76)	528 (78)	1.5 (-2.6, 5.7)	0.474
***Blood glucose monitoring and treatment***					
Venous blood glucose level sample collected and sent to laboratory		634 (82)	574 (85)	8.1 (1.5, 15)	**0.016**
Blood Glucose Level (BGL) monitored ≥ four times per day~	Day of admission^2^	591 (77)	530 (78)	5.7 (1.6, 9.9)	**0.007**
	Day two of admission^2^	407 (53)	472 (70)	20 (12, 27)	**< 0.001**
BGL ≥ 200 mg/dL within 48 h of admission		142 (19)	104 (15)	-3.6 (-7.8, 0.7)	0.100
Insulin given for first BGL ≥ 200 mg/dL		68 (53)	69 (67)	11 (-1.9, 24)	0.095
Insulin given within one hour from first BGL ≥ 200 mg/dL^2^		52 (41)	62 (60)	14 (1.1, 28)	**< 0.034**
Time from first BGL ≥ 200 mg/dL to insulin administration, mins (Median, (Q1, Q3))		14 (5, 59)	14 (0, 59)	-	0.3076
Monitored and treated according to the Hyperglycaemia (Sugar) Protocol^2^		319 (43)	375 (55)	23 (17, 29)	**< 0.001**
***Swallow screening***					
Formal swallow screen performed		532 (69)	510 (75)	15 (5.5, 24)	**0.002**
Failed swallow screen		74 (14)	83 (16)	0.68 (-3.6, 4.9)	0.755
Failed screen and subsequently had swallow assessment^3^		70 (95)	82 (99)	0.49 (-15, 16)	0.952
Swallow screen or assessment performed before being given oral medications^3^		506 (70)	481 (72)	2.7 (-1.6, 6.9)	0.222
Swallow screen or assessment performed before being given oral food or fluids^3^		478 (66)	472 (71)	4.1 (-0.3,8.4)	0.067
Swallow screen performed within 24hr^3^		485 (65)	500 (74)	18 (8.8, 26)	**< 0.001**
Monitored and treated according to the Swallow Protocol^3^		355 (52)	401 (61)	11 (5.2, 17)	**< 0.001**

For paracetamol and insulin administration outcomes, only patients with a documented fever or hyperglycaemic event or failed swallow screen within relevant time period were included in model. Denominator for swallow screen within 24 h; swallow screen or assessment before being offered food, fluids or medications is all patients

^ Estimated marginal mean difference in proportion (average marginal effects) from mixed effects model calculated using the STATA margins package with the dydx option, standard errors for confidence interval obtained using delta method

~ Only monitored if BGL unstable in first 48 h

# Must meet 1, 2 & 3 to be deemed as having been monitored and treated according to the complete FeSS Protocol

1 Must meet all elements to be deemed as having been monitored and treated according to the Fever Protocol

2 Must meet all elements to be deemed as having been monitored and treated according to the Hyperglycaemic (Sugar) Protocol

3 Must meet all elements to be deemed as having been monitored and treated according to the Swallow Protocol

Mixed effects logistic regression controlling for age, sex, NIHSS and correlation within hospital

Bold indicates *P* < 0.05; numbers may not add to total sample size due to missing values

## Discussion

Our primary outcome composite score results showed significant improvement from pre-to-post-implementation (pre 20%; post 28%, *p* < 0.001). However, there was room for improvement with less than a third of patients in the post-implementation cohort receiving care according to all of the FeSS Protocol elements. This is comparable to the findings of QASC Europe study, but whilst the results from 67 hospitals showed a lower pre-implementation adherence, they were able to achieve post-implementation adherence to all FeSS Protocol elements in approximately one third of patients (pre 3.6% vs. post 35%, *p* = 0.0365) [8]. This shows that delivering optimal stroke care encompassing all parameters targeted by the QASC protocol is a challenging endeavor. In Germany, it takes place before a backdrop of dire financial constraints faced by individual hospitals in combination with negative demographic trends of the nursing workforce. Both constitute a toxic environment for high quality nursing care. We would see this clinically meaningful and statistically significant improvement by 8% serving as a motivation to move forward.

Compared to other regions of the world [8, 14], we found high levels of measurements of body temperature pre-implementation; in > 95% of patients for day 1, 2 and 3, confirming high adherence to the OPS-requirement as we predicted. Conversely, measurement for elevated BGL and screening for dysphagia was less than optimally implemented pre-implementation with small but significant improvements at post-implementation. This underlines the importance of regular audit, feedback and education, and contradicts our initial assumption of remuneration being a key driver of performance. BGL management could be improved with an increase from 53 to 70% (adjusted difference 20%) of patients receiving regular BGL measurements on day 2 after admission; dysphagia management could be improved by increasing proportion of patients who received a standardized swallowing screen within 24 h after stroke from 65 to 74% of patients (adjusted difference 18%). These proportions are somewhat lower than data reported from the Stroke Registry of Northwestern Germany, who showed increasing proportions from 47.2 to 86.6% from 2008 to 2015 [15]. It is possible this discrepancy is because our data (75%) only include nurses’ performing swallowing screening and do not include any swallowing assessments performed by speech and language therapists.

Monitoring the vital parameters is only one part of the FeSS-Protocols, the other one being the therapeutic actions performed by the clinical teams as a result of abnormal parameters. In this aspect, we detected even greater changes: Against the background of excellent monitoring of body temperature, using the protocols lead to an adjusted increase of 32% in treating body temperature *≥* 37.5 °C within one hour with paracetamol. Similarly, treating hyperglycaemia was accelerated by giving insulin within one hour from first elevated glucose level (adjusted increase of 14%).

These data show that even against the background of tightly regulated stroke care in Germany, the nurse-led implementation of the FeSS Protocols resulted in measurable changes that can be summarized in a broader and more profound adherence to patient observation and goal-directed management of vital parameters. However, there remains space for improvement despite statistically significant increases.

When we set out to motivate German stroke units to participate in the QASC Germany project, there were doubts if there were any improvements to make, since the reimbursement requirements already demanded meticulous measurements and documentation of vital parameters which all had to be met for the composite measure. We assumed that this would lead to excellent results in vital parameter measurement and clinical consequences that would be hard to optimize. As we did not really expect changes, these clear results surprise all the more and give very clear indications for improving the process management in stroke care. Based on the experiences made in the QASC Germany project, the transfer of the responsibility for the process of monitoring and correcting these vital parameters to the nursing team should be considered, based on a protocol-based implementation strategy.

Our study was not designed to measure neurological outcome (e.g. disability 90 days after stroke), but we recorded stroke severity, pre-stroke disability and disability at discharge on mRS and the rate of hospital-acquired pneumonia. We noted statistically significant differences in the composition of the pre vs. post-implementation cohort in terms of pre-stroke disability (less patients with significant pre-stroke disability [mRS > 1] post-implementation) and stroke severity (more patients with NIHSS > 7 in the post-implementation cohort), which may be associated with pandemic related changes in patients, when patients with minor strokes tended to avoid hospitalization [16]. This clearly hampers the comparability of explorative outcome measures such as hospital-acquired pneumonia, disability at discharge and discharge status which should be interpreted with utmost caution. We did not see a statistically significant change in post-stroke pneumonia as a consequence of nurse-led dysphagia screening and the shorter nil-by-mouth times this possibly entrained.

While we were able to show a better adherence to guideline-based stroke care after the nurse-led implementation of the FeSS protocols, we cannot show effects on patient outcomes as our study was not designed to this end.

## Conclusions

The participating German stroke units showed good standards of monitoring the vital parameters after stroke [5, 8, 14], but there is still room for improvement. The nurse-initiated FeSS Protocols, supported by implementation strategies, increased the uptake of monitoring for the common post-stroke complications of fever, hyperglycaemia and swallowing difficulties and, as predicted, improved timeliness to treatment for the measures covered by the FeSS Protocol. However, the study confirms the importance of audit and feedback to support assumptions and not to rely on intuition. Next step will be the challenge to maintain and further improve these important stroke care processes beyond a one-time implementation.

## Data Availability

The datasets used and/or analysed during the current study are available from the corresponding author on reasonable request.

## Abbreviations

BGL: Blood Glucose Level
CI: Confidence Interval
DSG: Deutsche Schlaganfall-Gesellschaft/ German Stroke Society
EVT: Endovascular Stroke Therapy
FeSS: Fever Sugar Swallowing
INVN: Interdisziplinäres Neurovaskuläres Netzwerk/ Interdisciplinary Neurovascular Network
mRS: Modified Rankin Scale
NIHSS: National Institutes of Health Stroke Scale
OPS: Operationen und Prozeduren Schlüssel
Q: Quartile
QASC: Quality in Acute Stroke Care
SOP: Standard Operating Procedure
